# Simultaneous consumption of vegetable salad with bread attenuates postprandial serum glucose elevation in healthy adults: a single-ingestion open-label crossover trial

**DOI:** 10.1186/s13104-026-07836-0

**Published:** 2026-05-05

**Authors:** Mengwei Yuan, Naoki Kawada, Yumi Takeda, Ryosuke Matsuoka, Kazunori Utsunomiya

**Affiliations:** 1 R&D Division, Kewpie Corporation, 2-5-7, Sengawa-cho, Chofu, Tokyo 182-0002 Japan; 2 Jiseikai Nomura Hospital, 8-3-6, Shimorenja, Mitaka, Tokyo 181-8503 Japan

**Keywords:** Vegetable salad, Bread, Postprandial serum glucose, Crossover trial

## Abstract

**Objective:**

This study aimed to evaluate the effect of simultaneous consumption of vegetables and bread on postprandial serum glucose concentration. In total, 15 healthy men participated in this single-ingestion, open-label, non-randomized crossover trial. Participants were given meals (bread vs. bread with vegetable salad) after a night of fasting in a non-randomized sequence. At 0, 15, 30, 45, 60, 90, and 120 min following the consumption of the test meal, blood samples were collected to determine the serum levels of glucose, insulin, glucose-dependent insulinotropic polypeptide, and triglycerides.

**Results:**

Results revealed that serum glucose and insulin levels were significantly lower after 45 and 60 min in participants who consumed bread with vegetable salad than in those who only consumed bread. This emphasizes the potential benefit of simultaneously consuming vegetables and bread as an effective dietary strategy for preventing postprandial blood glucose elevation. Trial registration: UMIN Clinical Trials Registry (UMIN-CTR), UMIN000053931, registered on March 22, 2024.

## Introduction

In recent years, the prevalence of diabetes has been steadily increasing, emphasizing the importance of developing effective preventive strategies. Among the various contributing factors of diabetes, carbohydrate intake is considered a major cause of postprandial blood glucose elevation. In individuals with impaired glucose tolerance, postprandial hyperglycemia and insulin resistance may increase the risk of chronic diseases, including cardiovascular disorders [1–6].

The consumption of vegetable salads has been associated with a reduced risk of developing diabetes [7, 8]. This beneficial effect has been attributed to the physiological actions of bioactive components, such as vitamins, minerals, dietary fiber, and polyphenols, which are all commonly found in vegetables [9]. A study in Vietnam reported that the use of mayonnaise and dressings increased total vegetable intake, which was associated with reduced serum fructosamine levels—a clinical marker of glycemic control [10]. This suggests that consuming vegetables as “salads” with condiments enhances palatability and ease of intake, thereby increasing overall consumption and maximizing health benefits. Consequently, practical dietary strategies that promote higher vegetable intake are crucial for effective glycemic management.

The “salad-first” dietary approach, consuming salads before other dishes, reportedly suppresses rapid increases in postprandial blood glucose levels [11]. Numerous studies have investigated the regulatory effect of altering the order of food intake on glycemic responses [12–16]. However, many commonly consumed food items, such as sandwiches, pita wraps, and burritos, cannot be easily separated into different components. Therefore, the effect of consuming vegetable salads with carbohydrates in a single meal remains poorly evaluated. Thus, this study aimed to investigate the effects of the simultaneous consumption of a vegetable salad and bread on serum glucose concentrations.

## Materials and methods

### Study design

**Figure. 1:**
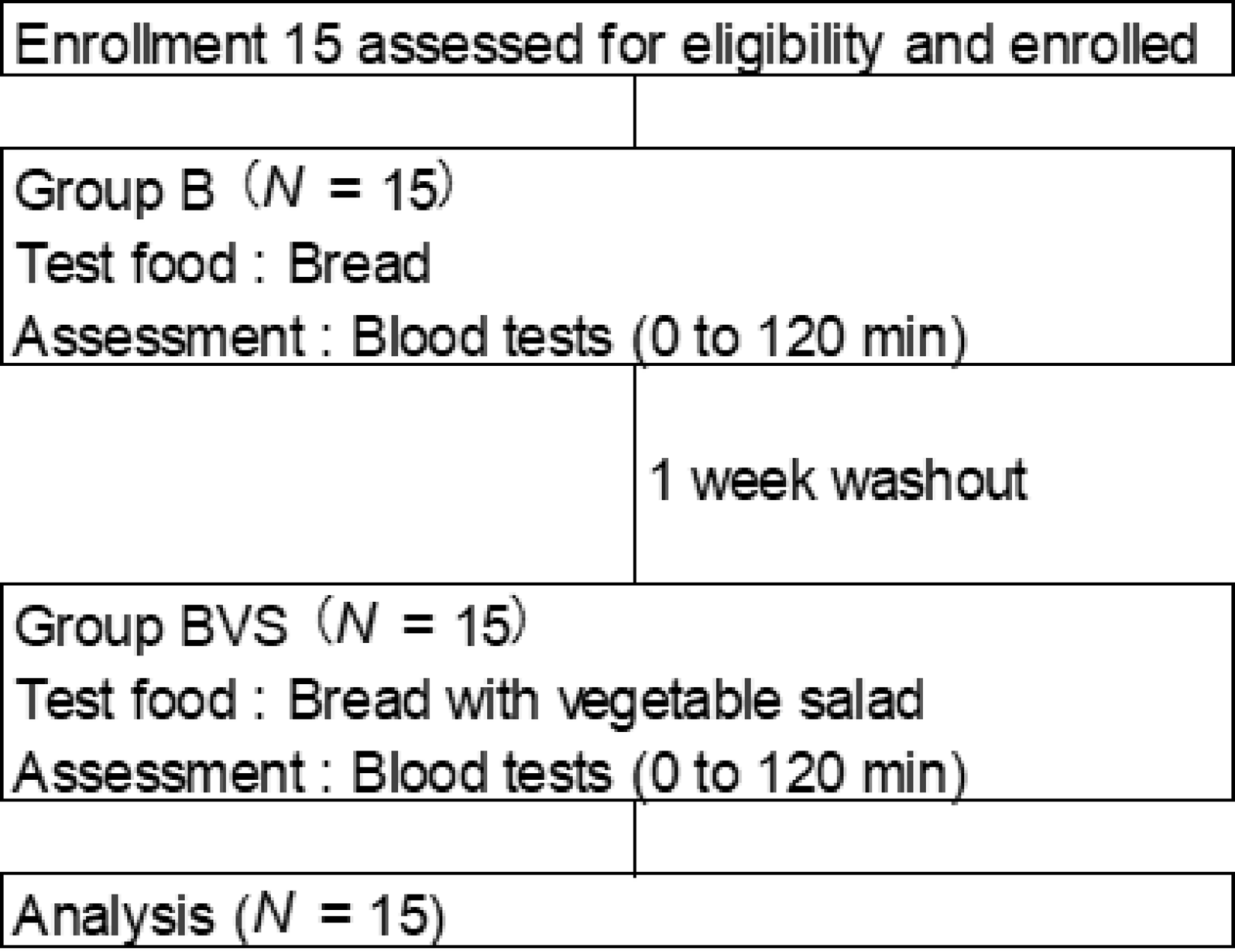
Participant flowchart

This single-ingestion, open-label, non-randomized crossover trial included 15 healthy men with a low risk of anemia from blood sampling. The flowchart of participant selection is shown in Figure. 1. Participants completed both test sessions without any dropouts. Thus, data from all 15 participants were included in the final statistical analysis. This study followed a non-randomized crossover design with a 7-day washout period between tests. All participants completed the trials in the same sequence, starting with bread alone (Group B) followed by bread with vegetable salad (Group BVS). The test meals were consumed within 10 min, with each mouthful chewed approximately 30 times. To standardize pre-test conditions, participants were instructed to maintain their usual lifestyle and refrain from overeating, excessive alcohol consumption, or strenuous physical activity on the day prior to each test. They were also required to fast from 9:00 PM until the start of testing the following morning. These measures were implemented to ensure a consistent baseline for postprandial measurements.

At 15, 30, 45, 60, 90, and 120 min after ingestion, blood samples were collected to measure the serum levels of glucose, insulin, glucose-dependent insulinotropic polypeptide (GIP), and triglyceride (TG). For fasting blood sampling, general tests for peripheral blood and serum analyses were performed.

This study only included participants who received a thorough explanation of the study protocol and provided written informed consent. Moreover, ethical approval was obtained from the Ethical Examining Committee of Ueno Asagao Clinic (Tokyo, Japan: Authorized No. 202401-39-KP001). This study was conducted at Hakunan Clinic (Chiba, Japan) and was registered at the University Hospital Medical Information Center (UMIN ID: UMIN000053931, Registered on 2024/03/22).

### Participants

Healthy male volunteers (aged 20–74 years) were recruited. Inclusion criteria were as follows: (1) no current medical treatment, (2) provision of written informed consent, and (3) willingness to maintain consistent lifestyle habits.

Exclusion criteria included the following: (1) current or past serious diseases (e.g., diabetes, cardiovascular, hepatic, renal disease, or cancer); (2) recent blood donation (within the past 1–3 months); (3) history of malaise during blood collection or difficult venous access; (4) severe anemia; (5) allergy to test meals; (6) regular use of health supplements; and (7) any other condition deemed inappropriate by the investigator. Eligibility was confirmed through initial screening, which assessed health status and baseline glycemic response.

### Test food

A vegetable salad (cabbage-based salad; Salad Club, Inc., Tokyo, Japan) and mayonnaise (Kewpie Corporation, Tokyo, Japan) were purchased commercially. Bread was prepared using a home bakery appliance in the Research and Development Division of Kewpie Corporation (Zojirushi BB-HA10, Zojirushi Corporation, Osaka, Japan). Table 1 enumerates the components of each test meal, and the nutrient composition of the vegetable salad and its extract.

**Table 1:** Composition and nutritional content of the test foods

		Bread	Bread with vegetable salad
Composition			
Bread (g)		70	70
Vegetable salad (g)		-	65
Mayonnaise (g)		-	15
Nutrient composition			
Energy (kcal)		185.5	299.3
Protein (g)		5.7	6.8
Fat (g)		2.9	14.2
Total carbohydrates (g)		35.7	39
Dietary fiber (g)		0	1.2

The value of the vegetable salad is included in roasted mayonnaise. Values are based on analytical data per serving

At breakfast, the B group received 70 g of bread, equivalent to one serving in Japan. Meanwhile, the BVS group consumed bread (70 g) and vegetable salad (65 g) mixed with mayonnaise (15 g). Considering that as little as 60 g of cabbage is reportedly effective in reducing blood glucose spikes, this study used 65 g of vegetable salad, consistent with the amount that is generally used in making sandwiches in daily diet [15]. Regarding the mayonnaise, the recommended amount per serving was used.

### Blood tests

Venous blood samples were collected via venipuncture at 0, 15, 30, 45, 60, 90, and 120 min. On the first test day, an additional 8.0 mL of blood was collected prior to the test meal for screening (6.0 mL for biochemical analysis and 2.0 mL for hematological analysis). At each postprandial time point, 8.0 mL of blood was drawn: 2.0 mL into a glucose tube for glucose and HbA1c, and 6.0 mL into specialized tubes for insulin, TG, and incretin measurements. In total, 62.0 mL of blood was collected on the first day and 56.0 mL on the second day.

For incretin measurement, samples were collected in EDTA tubes. For serum separation, blood was allowed to clot at room temperature and centrifuged at 3,500 rpm for 10 min. The resulting serum was stored under specific conditions: samples for glucose, insulin, and TG were kept at 2–8 °C, while samples for GIP analysis were immediately frozen at − 80 °C to ensure stability. Serum samples were analyzed for the specified parameters by LSI Medience Inc. (Tokyo, Japan): total cholesterol (enzyme method), low-density lipoprotein cholesterol (enzyme method), high-density lipoprotein cholesterol (direct method), TG (enzyme method), glucose (Hexokinase UV method), hemoglobin A1c (HbA1c; latex aggregation method), insulin (chemiluminescent enzyme immunoassay method), aspartate aminotransferase (AST), alanine aminotransferase (ALT; Japan Society of Clinical Chemistry [JSCC] transferable method), γ-glutamyl transpeptidase (γ-GTP; JSCC transferable method), urea nitrogen (urease-LED-UV method), creatinine (enzymatic method), uric acid (enzymatic method), and sodium, chlorine, and potassium (electrode method). Serum GIP levels were measured using enzyme-linked immunosorbent assay kit (Human GIP Total Assay Kit #27203, IBL Co. Ltd., Gunma, Japan).

### Sample size

Initially, the sample size was determined from previous studies of vegetable-first effects [14]. The Cancer Research and Biostatistics Statistical Tools software (https://stattools.crab.org/) was used for determining the sample size. The required sample size was 15 per group when α was 0.05 and power (1-β) was 80%.

### Statistical analysis

All results are expressed as mean ± SEM and median [interquartile range (IQR)]. The two groups were compared using the Wilcoxon signed-rank test and Bonferroni correction, as appropriate. The IQR was calculated as the difference between the 75th percentile (third quartile, Q3) and the 25th percentile (first quartile, Q1) of the data distribution. The same methods were used for comparing the serum levels of glucose, insulin, GIP, and TG with those before the ingestion of each of the meals. The incremental area under the curve (IAUC) for glucose, insulin, GIP, and TG was calculated using the trapezoidal rule. IAUC was defined as the area above the baseline concentration, obtained by summing the trapezoids formed between successive time points (0, 15, 30, 45, 60, 90, and 120 min). Results were expressed as mg·h/dL to reflect the cumulative postprandial response over the 2-hour period. The incremental maximum concentration (ΔCmax) was defined as the greatest change from baseline (0 min) for each participant during the 120-minute test. A p-value < 0.05 was considered statistically significant; all statistical analyses were performed using IBM SPSS Statistics version 29 (SPSS Japan Inc., Tokyo).

This study was conducted and reported in accordance with the CONSORT guidelines for non-randomized clinical trials.

## Results

### Participant demographics and hematology before test meal consumption

Table 2 presents the participants’ demographic characteristics. The body mass index was slightly high. All participants were healthy, with a mean age of 54 ± 2.1 years. The results of hematology, serum biochemistry, hepatic function, and renal function showed no significant differences before and after the consumption of either of the two meal types (*p* > 0.05).

**Table 2:** Participants’ demographic characteristics

	Participants		
Age (y)	54	±	2.1
Height (cm)	172.9	±	4.8
Body weight (kg)	76.7	±	6.8
BMI (kg/m²)	25.7	±	2.6
Body fat (%)	26.3	±	4.4
Systolic blood pressure (mmHg)	134.1	±	19.8
Diastolic blood pressure (mmHg)	90.9	±	17.2
Heart rate	74	±	12.6
Triglyceride (mg/dL)	150.5	±	87.1
HbA1c (%)	5.7	±	0.5
Glucose (mg/dL)	100.2	±	20.3
Insulin (µU/mL)	4.26	±	1.47

Mean ± SEM of 15 participants

### Changes in serum glucose and insulin levels

**Figure. 2:**
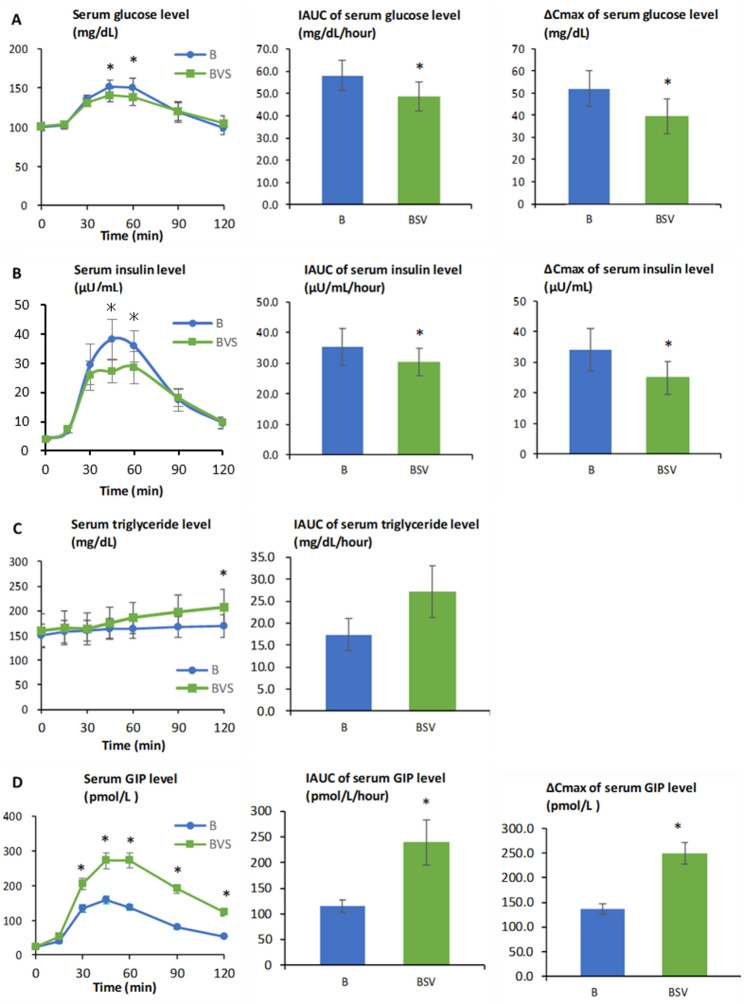
Postprandial serum glucose, insulin, triglyceride, and GIP responses after ingestion of bread alone (B) or bread with vegetable salad (BVS). **A** Serum glucose levels, incremental area under the curve (IAUC) of serum glucose levels, and changes in maximum concentration (ΔCmax) of serum glucose. **B** Serum insulin levels, IAUC of serum insulin levels, and ΔCmax of serum insulin. **C** Serum triglyceride levels. **D** Serum GIP levels, IAUC of serum GIP levels, and ΔCmax of serum GIP. Values are expressed as mean ± standard error of the mean (SEM). *p < 0.05 vs. B

Figure 2A and 2B, as well as Table 3, illustrate the changes, IAUC, and ΔCmax of serum glucose and insulin levels. At 45 and 60 min after test meal consumption, the serum glucose levels were significantly lower in the BVS group than in the B group (*p* < 0.05), indicating that the increase in serum glucose level was attenuated in the BVS group. Similarly, the IAUC (48.6 ± 6.7 vs. 58.1 ± 6.8 mg·h/dL, *p* = 0.023) and ΔCmax (141.2 ± 11.7 vs. 152.1 ± 12.5 mg/dL, *p* = 0.033) of the serum glucose levels were significantly lower in the BVS group than in the B group.

**Table 3:** IAUC and Cmax of blood glucose, insulin, triglyceride and incretin levels

Items		B (Mean ± SE / Median [IQR])	BVS (Mean ± SE / Median [IQR])	*p*
Blood glucose	IAUC (mg·h/dL)	58.1 ± 6.8 / 47.1 [47.1]	48.6 ± 6.7 / 39.6 [26.4]	0.023
	ΔCmax (mg/dL)	51.9 ± 8.1 / 57.0 [38.5]	39.5 ± 7.9 / 41.0 [16.0]	0.007
Insulin	IAUC (µU·h/mL)	35.3 ± 6.1 / 27.3 [25.9]	30.3 ± 4.5 / 23.9 [19.2]	0.027
	ΔCmax (µU/mL)	34.1 ± 6.9 / 29.1 [23.7]	24.9 ± 5.3 / 27.1 [23.7]	0.005
Triglyceride	IAUC (mg·h/dL)	17.3 ± 3.6 / 14.5 [12.6]	27.1 ± 5.9 / 26.6 [18.5]	0.080
GIP (pmol/L/hour)	IAUC (pmol/·h/L)	115 ± 12.1 / 117.8 [51.9]	238.7 ± 43.7 / 304.0 [166.4]	0.023
	ΔCmax (pmol/L)	136.8 ± 9.7 / 143.3 [59.1]	249.5 ± 21.4 / 273.2 [120.5]	0.000

Data are presented as mean ± SE and median ± IQR (*n* = 15). Statistical significance was determined using the Wilcoxon signed-rank test. IQR represents the interquartile range (75th percentile minus 25th percentile)

*p < 0.05 vs. B

### Changes in serum TG levels

As shown in Figure 2C and Table 3, the serum TG levels were significantly higher in the BVS group than in the B group at 120 min after test meal consumption. The IAUC for TG is also presented in Table 3.

### Changes in serum GIP levels

As shown in Figure 2D and Table 3, the serum GIP levels as well as the IAUC were significantly higher in the BVS group than in the B group at 30–120 min following meal consumption.

## Discussion

This study demonstrated that the simultaneous consumption of vegetable salad and carbohydrate-rich foods, such as bread, attenuates the postprandial elevation of serum glucose levels (Figure 2A). Although the “salad-first” approach—consuming vegetables before carbohydrate-rich foods—has been effective in moderating postprandial glycemic responses, reports on the effects of concurrent intake, particularly in the context of composite meals (e.g., sandwiches or burritos) where sequential consumption is impractical, remain limited. Results of the current study suggest that the simultaneous intake of vegetables and carbohydrates can be beneficial in blunting glycemic excursions, offering a more feasible dietary strategy for daily life.

Cabbage, the primary component of the salad used in this study, is rich in insoluble dietary fiber, specifically cellulose. This type of fiber increases the viscosity of gastrointestinal contents and physically impedes the diffusion of nutrients and digestive enzymes, potentially slowing carbohydrate digestion and absorption [17–19]. Furthermore, cabbage contains rutin, a polyphenol that inhibits α-glucosidase activity and improves glucose metabolism, contributing to the suppression of carbohydrate breakdown and absorption [20].

Moreover, the salad was mixed with mayonnaise, which contains dietary fats that prolong gastric emptying time, thereby delaying the transfer of nutrients to the small intestine [21]. The acetic acid present in mayonnaise—derived from vinegar—can also suppress postprandial glycemic elevation by delaying gastric emptying and potentially enhancing insulin sensitivity [22, 23]. These combined effects likely contributed to the observed reduction in postprandial serum glucose levels. Importantly, the fat in the mayonnaise used in this study was primarily canola oil, which is rich in high-quality unsaturated fatty acids. The glycemic-modulating effects observed are therefore associated with the intake of these unsaturated fats. Importantly, the choice of fatty acid type is critical for metabolic health; compared with saturated fatty acids, which may impair insulin sensitivity, unsaturated fatty acids (particularly monounsaturated fats found in vegetable oils) are reported to exert more favorable effects on postprandial glycemic control and insulin secretion [24]. Therefore, minimizing saturated fat intake while incorporating high-quality unsaturated fats is a recommended strategy for mitigating postprandial glucose spikes without increasing cardiovascular risk [25]. Regarding the lipid response, the BVS group exhibited significantly higher serum TG levels than the B group toward the end of the 120-minute period. This difference is explained by the slower digestion and absorption of dietary lipids compared with carbohydrates. Plasma TG levels typically peak 3–6 h after a meal, as lipids are transported via chylomicrons into systemic circulation [26]. While the inclusion of these fats contributed to the prolonged TG elevation, it also played a crucial role in attenuating the postprandial glucose spike by delaying gastric emptying. Our findings, showing a steady rise in TG levels up to 120 min, are consistent with established kinetics of postprandial lipid metabolism.

Interestingly, despite the elevated serum GIP levels observed in the BVS group, the serum glucose and insulin levels were lower than those in the B group. GIP is secreted from K-cells in the upper small intestine in response to dietary carbohydrate and fat intake, and acts on pancreatic β-cells to promote insulin secretion [26, 27]. However, GIP exerts its insulinotropic effects in a glucose-dependent manner; when blood glucose levels are low or only mildly elevated, GIP cannot substantially stimulate insulin secretion. Indeed, Meier et al. and Nauck et al. reported that elevated GIP concentrations do not necessarily result in enhanced insulin secretion when glycemic levels remain low [27, 28]. The present findings align with this physiological mechanism. Therefore, enhanced GIP secretion, combined with modest postprandial glycemia, may support glucose regulation without excessive insulin demand.

Thus, these findings suggest that glycemic response reduction observed in this study is likely attributable to the combined effects of dietary fiber, polyphenols, fat, and acetic acid. These components may act synergistically to slow the digestion and absorption of nutrients while modulating the secretion of incretin hormones. Notably, effective glycemic control was achieved without the need to consume vegetables prior to carbohydrates, indicating such simultaneous intake as a metabolically favorable strategy. This dietary approach is practical and sustainable for busy individuals who may find strict food sequencing difficult to maintain. Consistent with these results, Uenaka et al. reported that the combination of dietary fiber, vinegar, and fat produced a greater glycemic-lowering effect when consumed with rice, compared with any of these components alone [29]. This finding supports the notion that multicomponent dietary strategies can act synergistically to enhance postprandial glycemic regulation, especially in typical mixed-meal settings.

While this study focused on blood-based metabolic markers, particularly incretin responses, the direct physical effects of the mixed meal on digestion remain to be clarified. The synergistic interaction between emulsified lipids in mayonnaise and insoluble fiber in cabbage likely influences gastric viscosity and phase separation. Future studies should therefore employ in vitro or in vivo gastric digestion simulations to assess nutrient disintegration rates and enzyme accessibility. Such simulation-based approaches would provide a more mechanistic understanding of how simultaneous intake physically modulates carbohydrate digestion kinetics. To further enhance postprandial glycemic regulation under realistic dietary conditions, future research should focus on identifying the optimal combinations of vegetables, dietary fibers, polyphenols, and accompanying condiments or fats.

## Conclusions

The postprandial increase in serum glucose level was suppressed when bread was consumed simultaneously with vegetable salad and mayonnaise. This effect is likely attributable to multiple factors, including the dietary fiber and bioactive compounds contained in the vegetables as well as the fat and acetic acid present in the mayonnaise.

### Limitations

In this study, the individual effects of vegetable salad and mayonnaise were not evaluated separately; therefore, it was not possible to determine which component contributed to the observed outcomes. Moreover, the actual rate of digestion within the gastrointestinal tract was not directly measured, limiting insight into the temporal dynamics of digestion and absorption.

To clarify the underlying mechanisms, future studies should evaluate the biokinetics of the target components, particularly their absorbability and receptor-binding capacity in the small intestine.

## Data Availability

The data presented in this study are available on request from the corresponding author.

## Abbreviations

BVS: Bread with vegetable salad
BMI: Body mass index
HbA1c: Hemoglobin A1c
GIP: Glucose-dependent insulinotropic polypeptide
IAUC: Incremental area under the curve
JSCC: Japan society of clinical chemistry
